# Anthropometric measures and the risk of coronary artery disease

**DOI:** 10.22088/cjim.11.2.183

**Published:** 2020

**Authors:** Aidin Baghbani-Oskouei, Mehrzad Gholampourdehaki

**Affiliations:** 1 Department of Internal Medicine, School of Medicine, AJA University of Medical Sciences, Tehran, Iran

**Keywords:** Coronary artery disease, Anthropometric, Body mass index, Waist to hip ratio, Wrist circumference

## Abstract

**Background:**

Nowadays, obesity and overweight are projected to become main risk factors for coronary artery disease (CAD). We aimed to determine the association of anthropometric measures with presence of significant (sig.) CAD as evaluated by coronary angiography, among an adult Iranian population.

**Methods:**

The present study included 441 patients (men=275) aged > 30 years with suspected CAD, who had undergone coronary angiography between January 2019 and November 2019. All demographic data and patients’ medical history as well as clinical examinations were recorded by a trained physician. Coronary angiography was performed using standard techniques to determine the presence of sig. coronary artery lesions. Logistic regression analyses were conducted to assess the odds ratio (OR) of each anthropometric measure for the presence of sig. CAD.

**Results:**

The mean age of participants was 51.2±8.7 years and sig. CAD was detected in 185 patients. Univariate analyses showed that body mass index (BMI), waist circumference (WC), and waist to hip ratio (WHR) were significantly associated with increased risk of CAD. On multivariable logistic regression model, BMI and WHR correlated independently with increased risk of CAD; while higher WC and wrist circumference (WrC) could not predict the CAD risk. The corresponding ORs (95% confidence interval) were 1.36 (1.04-1.74), 1.17 (0.95-1.63), 1.29 (1.12-1.41), and 1.24 (0.76-1.92) for BMI, WC, WHR, and WrC, respectively. Considering the receiver operating characteristic analysis, no superiority was observed for each of the measures for discriminating sig. CAD from non-sig. CAD status.

**Conclusion:**

BMI and WHR are independently associated with the presence of CAD among Iranian population. These results emphasize the value of anthropometric assessment among those with suspected CAD.

Nowadays cardiovascular diseases (CVD) have been regarded as a major cause of mortality and morbidity in both the developed and developing nations (1). World Health Organization (WHO) has recently reported that in the future, the global burden of cardiovascular mortality is mostly estimated to occur in the developing countries (1); thus, population-based multi-ethnic studies are warranted to identify the underlying risk factors for CVD. The incidence rates of coronary heart disease in Iran were estimated as 16.8 and 9.8 per 1000 person‐years among men and women, respectively (2). Of the different potential risk factors, the impacts of obesity and anthropometric factors on developing coronary artery disease (CAD) have been clearly identified by the American Heart Association and Framingham Heart study (3, 4).

The incidence of obesity is rising in the developing countries, probably due to the sedentary lifestyle and Westernization of dietary manner (5); the prevalence of obesity was estimated to be more than 50% in a recent review study on Middle-Eastern population (6). Although body weight and body mass index (BMI) are determined as main indices for overweight and obesity, they seemed to fail to differentiate fat from muscle mass and cannot show the body’s fat distribution pattern, as well. On the other hand, central fat distribution is considered more atherogenic than peripheral obesity (7); thus, some other anthropometric measures including waist circumference (WC) and waist to hip ratio (WHR) have been suggested which were proven to raise the risk of cardio-metabolic diseases and mortality, independent of patients’ weight (8, 9). Recently, some other structural parameters of the body such as wrist circumference (WrC) have been also introduced for predicting CVD (10).

Regarding the high prevalence and incidence rates of both CVD and obesity in Iranian population (11, 12) and considering controversies in the correlation between anthropometric parameters and CAD in several previous studies, we aimed to extend previous observations by investigating the associations of anthropometric measures with the presence of significant (sig.) CAD among an adult Middle-Eastern population.

## Methods

**Study population: **This is a case-control study conducted in Imam Ali Hospital and Mahallati Hospital, Tabriz, Iran from January 2019 to November 2019. A total of 543 clinically stable patients aged > 30 with suspected CAD, who were candidate for undertaking coronary angiography by interventional cardiologists, were screened for enrolment and after exclusion of patients with prior history of revascularization by percutaneous or surgical interventions (n=72) and those with major concomitant non-CVD [e.g. severe congestive cardiac failure (n=14), chronic kidney disease (n = 10), and chronic systemic inflammatory disease (n=6)] 441 were enrolled in the study.

All participants read and signed an informed consent form and the Ethics Committee of AJA University of Medical Sciences approved the study design and protocol, in agreement with the Declaration of Helsinki Guidelines.

**Clinical and laboratory measurements: **A trained interviewer collected all demographic data and participants’ medical history using a pretested questionnaire. The investigated demographic data contained age, gender, educational status, smoking habit, the presence of comorbid conditions (i.e. hypertension, type 2 diabetes mellitus, congestive cardiac failure, chronic inflammatory disease, chronic kidney disease), and prior history of coronary artery revascularization by percutaneous or surgical interventions. Patients were assessed in an examination gown, with upper body clothing and shoes removed. All of them had a resting time for at least 20 min before measuring their right arm blood pressure in a sitting position which was done by a desk-model sphygmomanometer.

Body height and weight were measured to compute BMI. Weight was measured using digital electronic weighing scale and rounded to the nearest 100g. Height was evaluated using a tape meter; patients were in standing position and the shoulders were in normal alignment. BMI= body weight (kg)/ height^2^ (m^2^). For the WHR, the waist is measured at the level of umbilicus, and the hip circumference was measured at the level of the buttocks’ greatest protuberance and widest area of the hip; WHR = waist measurement (cm)/ hip measurement (cm). WrC was measured by an inflexible tape meter, positioned over the distal of ulna and radius (Lister's tubercle).

A blood sample was collected from all patients between 6∶30 and 10∶00 AM (after 9 to 11 hours overnight fasting status). All blood tests were carried out at the Imam Ali Hospital’s laboratory on the blood sampling day. By using standard methods on a Cobas auto-analyzer system, samples were assayed for fasting plasma glucose (FPG) and lipid profile including total cholesterol, triglycerides (TG), low-density lipoprotein cholesterol (LDL-C), and high-density lipoprotein cholesterol (HDL-C). Serum creatinine was assayed using the photometric Jaffe method.

During coronary artery angiographic evaluations, the degree of stenosis was quantified by visual assessment of the reduction in diameter of the lumen relative to the adjoining normal segment of vessel while moving cineangiogram.

**Definition of terms: **Education was categorized into two groups, as stated by participants: no formal education and some formal education. A current smoker was defined as smoking cigarettes daily or occasionally. The diagnosis of diabetes mellitus was defined as taking anti-diabetic medications or meeting one or both of these criteria: FPG ≥ 7 mmol/L, and 2-hour postprandial glucose ≥ 11.1 mmol/L. The diagnosis of hypertension was defined as using anti-hypertensive drugs or based on systolic blood pressure (SBP) and diastolic blood pressure (DBP) measurements: SBP ≥ 140 mmHg or DBP ≥ 90 mmHg.

For excluding purposes, experiencing any acute coronary syndrome or myocardial infarction, or undergoing coronary angiography was defined as prior history of CAD. Patients were considered as positive family history of CVD if there were any previous diagnosis of CVD in first-degree male relatives, aged ˂ 55 years or first-degree female relatives, aged ˂ 65 years. Severe congestive cardiac failure was defined as a cardiac functional classification of III or IV as determined by the New York Heart Association (NYHA) criteria. Systemic inflammatory disease was defined as the presence of one of following diseases: rheumatoid arthritis/polyarthritis, polymyalgia rheumatica, polymyositis, dermatomyositis, giant cell arteritis, systemic lupus erythematosus, Sjoegren’s syndrome, systemic sclerosis, and spondylitis ankylosing. Chronic kidney disease was defined based on the Kidney Disease Outcome Quality Initiative guideline, as either kidney injury or estimated glomerular filtration rate (eGFR) < 60 mL/min/1.73 m^2^ for more than three months. In the current study, eGFR was measured using abbreviated prediction equation made available by the Modification of Diet in Renal Disease (MDRD) study (13).

Patients were categorized into two groups, according to the angiographic documentations: The sig. CAD group, if there were meaningful coronary artery involvement (equal or greater than 50% luminal stenosis) in one major epicardial coronary artery (i.e. left anterior descending, circumflex, or right coronary artery) or their branches with diameter of at least 2.5 mm; and non-sig. CAD group, if there was not any evidence of sig. coronary stenosis or the presence of stenosis less than 50%.

**Statistical Analyses: **Kolmogorov–Smirnov test was performed for the presence of normal distribution for all numeric variables. Normally distributed continuous variables were declared as the mean and standard deviation (SD), and skewed variables were described as median and interquartile range (IQR) 25^th^–75^th^. Additionally, we presented categorical variables as number and percentage (%). To test for differences in the baseline characteristics between sig. CAD and non-sig. CAD groups, independent-samples t-test, Mann-Whitney test, or Pearson's Chi-squared test was performed, as appropriate.

We conducted logistic regression analyses to estimate the odds ratios (ORs) and their 95% confidence interval (CI) of contributing risk factors as independent variables for the presence of sig. CAD, as the dependent variable. We fitted both unadjusted and multivariate adjusted models; the relevant ORs and 95% CI were reported. We furtherly analyzed the receiver operating characteristic (ROC) curve for determining the value of each anthropometric measure for discriminating sig. CAD from non-sig. CAD status. A good discriminative value was defined as area under curve (AUC) of more than 0.80. We also compared the predictive power of different anthropometric measures, as assessed by the AUC, to discriminate sig. CAD from non-sig. CAD (without adjustment for any covariate).

Statistical analyses and data processing were performed by STATA (Version 14) and SPSS (Version 20) programs for windows. A p value below 0.05 considered to indicate statistically significance.

## Results

A total of 441 individuals (men=275) with mean age of 51.2±8.7 years, were recruited for the currentstudy. The most common comorbidity was hypertension (78.9%) followed by diabetes mellitus (22.2%); the prevalence of family history of CVD and cigarette smoking was 39.5 and 31.1%, respectively. Table 1 illustrates the baseline characteristics of the participants with and without CAD. 

To compare the two groups, subjects with CAD were older and presented higher male gender and current smoker frequencies, higher mean SBP, DBP, BMI, WC, WHR, as well as higher prevalence of hypertension. Furthermore, they had higher levels of FPG and TG and lower level of HDL-C. However, there was no statistically sig. difference in terms of educational status, WrC, TC, LDL-C, and creatinine levels, prevalence of diabetes mellitus, and family history of CVD between the two groups. Moreover, univariate analyses along with the OR and 95% CI of contributing risk factors for developing CAD are shown in table 1. 

Accordingly, of anthropometric measures, BMI, WC, and WHR were significantly associated with increased risk of CAD; the corresponding ORs (95% CI) were 1.71 (1.08-2.29), 1.34 (1.04-2.18), and 1.36 (1.21-1.52), respectively. However, there was no statistically sig. association between WrC and the sig. CAD risk (*P*=0.208).

**Table 1:** Characteristics of the study population with and without significant coronary artery disease

	**Non-sig. CAD group** **(n = 256)**	**Sig. CAD group** **(n = 185)**	**Total** **(n = 441)**	**Odds ratio** **(95% CI)**	***P*** ** value**
Male gender, n (%)	152 (59.3)	123 (66.5)	275 (62.4)	1.67* (1.10-2.15)	0.005
Age (years), mean (SD)	48.4 (8.4)	56.8 (9.3)	51.2 (8.7)	1.87* (1.09-3.01)	0.019
Education, n (%)					0.345
No formal education	58 (22.7)	45 (24.3)	96 (21.8)	1.00 (reference)	
Some formal education	198 (77.3)	140 (75.7)	345 (78.2)	0.95 (0.61-1.54)	
Current smoker, n (%)	65 (25.4)	72 (38.9)	137 (31.1)	1.48* (1.10-1.98)	0.012
Diabetes mellitus, n (%)	49 (19.1)	39 (21.0)	98 (22.2)	1.39 (0.81-2.17)	0.213
Hypertension, n (%)	192 (75.0)	156 (84.3)	348 (78.9)	1.63* (1.05-2.53)	0.025
Family history of CVD, n (%)	97 (37.9)	77 (41.6)	174 (39.5)	1.21 (0.89-2.04)	0.198
SBP (mmHg), mean (SD)	136.9 (11.1)	142.9 (9.6)	139.4 (10.5)	1.06* (1.02-1.26)	0.006
DBP (mmHg), mean (SD)	83.5 (7.4)	85.8 (6.3)	84.5 (6.9)	1.17* (1.09-1.51)	0.041
BMI (Kg/m^2^), mean (SD)	25.8 (3.9)	27.3 (4.2)	26.4 (4.0)	1.71* (1.08-2.29)	0.009
WC (cm), mean (SD)	86.7 (12.1)	91.3 (10.8)	88.6 (11.6)	1.34* (1.04-2.18)	0.017
WHR, mean (SD)	0.89 (0.006)	0.94 (0.007)	0.92 (0.006)	1.36* (1.21-1.52)	< 0.001
WrC (cm), mean (SD)	17.6 (1.01)	17.8 (0.99)	17.7 (1.00)	1.44 (0.85–2.44)	0.208
FPG (mmol/L), median (IQR)	4.88 (4.60-5.21)	4.99 (4.71-5.32)	4.93 (4.60-5.27)	1.54* (1.02-2.37)	0.041
TC (mmol/L), mean (SD)	4.25 (0.08)	4.29 (0.08)	4.27 (0.08)	1.19 (0.66–2.86)	0.685
TG (mmol/L), median (IQR)	1.36 (0.95-2.02)	1.68 (1.17-2.39)	1.50 (1.03-2.16)	2.10* (1.29–3.41)	0.028
LDL-C, mmol/L), mean (SD)	2.69 (0.93)	2.92 (1.00)	2.79 (0.96)	1.24 (0.99-1.56)	0.058
HDL-C (mmol/L), mean (SD)	1.11 (0.13)	0.88 (0.12)	1.02 (0.13)	0.71* (0.41-0.87)	< 0.001
Creatinine (μmol/L), mean (SD)	104.3 (32.5)	101.8 (28.7)	103.3 (30.9)	1.21 (0.71-1.84)	0.442

* Significant

Sig., significant; CAD, coronary artery disease; CI, confidence interval; n, number; SD, standard deviation; SBP, systolic blood pressure; DBP, diastolic blood pressure; CVD, cardiovascular disease; BMI, body mass index; WC, waist circumference; WHR, waist to hip ratio; WrC, wrist circumference; FPG, fasting plasma glucose; IQR, interquartile range; TC, total cholesterol; TG, triglycerides; LDL-C, low density lipoprotein cholesterol; HDL-C, high density lipoprotein cholesterol.

Variables associated with CAD risk on univariate analysis were considered for the multivariate model analysis. On multivariable logistic regression model, male gender, age, smoking, hypertension and higher SBP, and lower level of HDL-C increased the risk of sig. CAD; the corresponding ORs (95% CI) were 1.52 (1.09-1.98), 1.69 (1.07-2.31), 1.39 (1.04-1.79), 1.04 (1.02-1.21), and 0.79 (0.56-0.91) for gender (male), age, smoking, hypertension, SBP, and HDL-C, respectively. 

Table 2 presents the independent OR of different anthropometric measures for sig. coronary stenosis. Accordingly, BMI and WHR were significantly associated with 36 and 29% increased risk of CAD, respectively; while higher WC and WrC could not predict the CAD risk. The corresponding ORs (95% CI) were 1.36 (1.04-1.74), 1.17 (0.95-1.63), 1.29 (1.12-1.41), and 1.24 (0.76-1.92) for BMI, WC, WHR, and WrC, respectively.

**Table 2:** Association of anthropometric measures and significant coronary artery stenosis: results of multivariable logistic regression analysis

**Factors**	**OR**	**95 % CI for OR**		***P*** ** value**
		**Lower**	**Upper**	
BMI	1.36*	1.04	1.74	0.027
WC	1.17	0.95	1.63	0.083
WHR	1.29*	1.12	1.41	0.006
WrC	1.24	0.76	1.92	0.435

* Significant

OR, odds ratio; CI, confidence interval; BMI, body mass index; WC, waist circumference; WHR, waist to hip ratio; WrC, wrist circumference.

**Figure 1:**
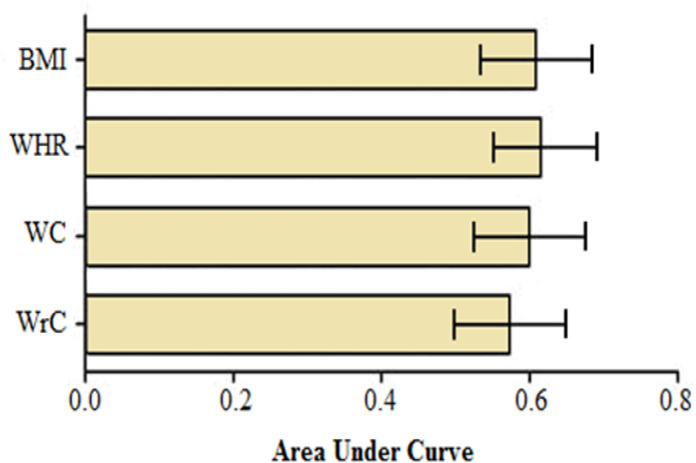
Area under the receiver operating characteristic curve and 95% confidence interval of the anthropometric measures for discriminating sig. CAD from non-sig. CAD status. The anthropometric variables were BMI, WHR, WC, and WrC. Error bars indicate 95% confidence interval. The area under the curve for BMI, WHR, WC, and WrC are 0.606, 0.615, 0.598, and 0.574, respectively

Figure 1 highlights the ROC curve analysis of anthropometric measures for discriminating CAD from non-CAD status. Accordingly, none of the anthropometric measures, including BMI, WHR, WC, and WrC, could discriminate CAD from non-CAD subjects. Considering discriminatory abilities as assessed by the AUC, WHR seemed to have the highest power (≈ 62%), but statistically no superiority was observed for WHR compared with BMI and WC; the AUC for BMI, WHR, and WC were almost similar but higher than those for WrC (*P*˂0.05). 

## Discussion

Anthropometric measures have been determined as important measurements for risk assessment of cardio-metabolic diseases, according to the results of large studies such as the well-known Framingham survey (14). Controversy regarding the correlation between anthropometric measures and CAD in several previous studies, was the motivation for us to conduct the current study; we examined the impacts of anthropometric measures on the risk of CAD among an adult Iranian population. Accordingly, higher BMI and WHR were found to associate with 36% and 29% increased risk of CAD, respectively; however, there was no superiority for each of anthropometric measures for CAD discrimination.

Generally, WC and WHR are used as measures of central or visceral obesity, while BMI indicated overall obesity. Furthermore, a large Australian study proposed that the hip circumference determines a lower risk for body fat accumulation, so including it in the WHR equation increases the accuracy of measurement (15). Several investigations demonstrated superiority of WHR as a better risk factor for CAD than WC per se and BMI (16-19). Lakka et al. in a cohort study on 1346 middle-aged men, being followed for 10.6 years, reported that WHR, WC, and BMI directly increased the CAD risk, and the WHR adds to the value of BMI in coronary accidents prediction; however, BMI could not provide additional predictive value beyond WHR (20). Several studies in Iran, Pakistan, and Kosovo showed that WHR had a positive correlation with the risk of severe CAD (21-23). Sabah et al. in another study highlighted the superiority of WHR than BMI for developing CAD and yielded that patients with BMI ≥ 25 Kg/m^2^ and WHR ≥ 0.55 are 3.06 and 6.77 times, more likely to develop sig. CAD, respectively (18). Similarly, Zen et al. found that BMI ≥ 30 Kg/m^2^ vs. ˂ 25 Kg/m^2^ increased 2.3 times the chance of sig. coronary stenosis; and men with WHR ≥ 0.85 and women with WHR ≥ 0.95 showed 4.0 times higher risk for CAD even after controlling for confounding factors, including BMI [compared to those with WHR ˂ 0.80 (men) and ˂ 0.90 (women)] (24). In concordance to these findings, some studies found an inverse relationship between BMI and the risk and severity of CAD (22, 25, 26). Morricone et al. proposed that the severity of CAD is correlated with WHR in nondiabetic patients with normal weight, but showed a negative association with BMI particularly among nondiabetic obese subjects (27).

Taken together, these results emphasize the value of anthropometric assessment among those with suspected CAD, although the role of fat distribution on CAD risk should be clarified. It seems that visceral or central adipose tissue is metabolically more active and pathological than subcutaneous adipose tissue by inducing immunity processes, which leads to atherosclerotic CVD (28-30). In other words, atherosclerotic disease does not result from the adipose tissue accumulation per se, but is as a result of adipose tissue dysfunction or ‘sick fat’ (30). Although we found no difference between the power of general and central obesity variables for identifying CAD, it seems that ethnical differences may have an important effect and can alter the power of anthropometric measures in predicting CAD, as a possible confounder. The contribution of smoking, hypertension, diabetes, and lipid profiles are also variable between different studies. Furthermore, there can be inaccuracy in self-reported measurements in some studies that can cause invalid results. Insufficient or over-adjustment of confounders and other cardiovascular risk factors also may have important role in discovering the nature of this association.

Recently, WrC considered as peripheral fat distribution index, has attracted more attention. It has been demonstrated as an important cardio-metabolic risk factor in a meta-analysis study (31), which can help as a simple clinical marker to identify high risk subjects. Mohebi et al. in a cohort study in 2014, highlighted the role of WrC as a novel anthropometric measure, in predicting CVD incidence among non-obese women, although it failed to predict CVD events in centrally obese women (WC ≥ 95 cm) (10). In the current study, at the first step, CAD subjects were seemed to have higher WrC. However, in logistic regression analyses, we did not find any association between WrC and the risk of CAD, and overall predictive discrimination for BMI, WHR, and WC (as judged by AUC) was better than WrC. Similar to our results, Hajsadeghi et al. suggested that WrC could not have a predictive value for the presence of CAD (32). Further studies are needed to explore this relation and the underlying potential mechanisms.

To the best of our knowledge's this is the first study to assess the impacts of BMI, WC, WHR, and WrC simultaneously on CAD risk. Efforts were made to reduce possible biases; a single interviewer collected the data throughout the study period and ensured quality control and corrections for eliminating the inter-observer and intra-observer variations. There are some limitations for this study. First, the cross-sectional design; and the second, small number of CAD cases, which might reduce the power of the statistical analyses. Unfortunately, in the current study we did not ask about the patients’ duration of being smoker, hypertensive, and diabetic, which might be more important than the presence of each condition. Finally, current study’s control group included patients who were referred for conventional coronary angiography at the discretion of their cardiologist and is not a true representative of general population; thus, extrapolation of the results to other populations would be unwise.

We found BMI and WHR as independent risk factors for the presence of sig. CAD among an Iranian population, which could aid in coronary risk assessment in the general clinical setting. Furthermore, given the high prevalence and incidence of CVD among Iranain population (33), primary prevention and intervention programs designed to reduce obesity through lifestyle modification, i.e. diet and physical activity may have serious public health implications in preventing CAD.

## References

[1] Oliveira GB, Avezum A, Roever L (2015). Cardiovascular disease burden: evolving knowledge of risk factors in myocardial infarction and stroke through population-based research and perspectives in global prevention. Front Cardiovasc Med.

[2] Kabootari M, Asgari S, Mansournia MA (2018). Different weight histories and risk of incident coronary heart disease and stroke: tehran lipid and glucose study. J Am Heart Assoc.

[3] Eckel RH (1997). Obesity and heart disease: a statement for healthcare professionals from the Nutrition Committee, American Heart Association. Circulation.

[4] Hubert HB, Feinleib M, McNamara PM, Castelli WP (1983). Obesity as an independent risk factor for cardiovascular disease: a 26-year follow-up of participants in the Framingham Heart Study. Circulation.

[5] Zhang Y, Hong J, Gu W (2009). Impact of the metabolic syndrome and its individual components on risk and severity of coronary heart disease. Endocrine.

[6] Ng M, Fleming T, Robinson M (2014). Global, regional, and national prevalence of overweight and obesity in children and adults during 1980–2013: a systematic analysis for the Global Burden of Disease Study 2013. Lancet.

[7] Siavash M, Sadeghi M, Salarifar F, Amini M, Shojaee-Moradie F (2008). Comparison of body mass index and waist/height ratio in predicting definite coronary artery disease. Ann Nutr Metab.

[8] Zhang C, Rexrode KM, van Dam RM, Li TY, Hu FB (2008). Abdominal obesity and the risk of all-cause, cardiovascular, and cancer mortality. Circulation.

[9] Czernichow S, Kengne AP, Stamatakis E, Hamer M, Batty GD (2011). Body mass index, waist circumference and waist–hip ratio: which is the better discriminator of cardiovascular disease mortality risk? Evidence from an individual‐participant meta‐analysis of 82 864 participants from nine cohort studies. Obes Rev.

[10] Mohebi R, Mohebi A, Sheikholeslami F, Azizi F, Hadaegh F (2014). Wrist circumference as a novel predictor of hypertension and cardiovascular disease: results of a decade follow up in a West Asian cohort. J Am Soc Hypertens.

[11] Khalili D, Sheikholeslami FH, Bakhtiyari M (2014). The incidence of coronary heart disease and the population attributable fraction of its risk factors in Tehran: a 10-year population-based cohort study. PloS One.

[12] Hosseinpanah F, Mirbolouk M, Mossadeghkhah A (2016). Incidence and potential risk factors of obesity among Tehranian adults. Prev Med.

[13] Levey AS, Greene T, Kusek JW, Beck GJ (2000). A simplified equation to predict glomerular filtration rate from serum creatinine. J Am Soc Nephrol.

[14] Preis SR, Massaro JM, Hoffmann U (2010). Neck circumference as a novel measure of cardiometabolic risk: the Framingham Heart study. J Clin Endocrinol Metab.

[15] Welborn TA, Dhaliwal SS (2007). Preferred clinical measures of central obesity for predicting mortality. Eur J Clin Nutr.

[16] Gruson E, Montaye M, Kee F (2010). Anthropometric assessment of abdominal obesity and coronary heart disease risk in men: the PRIME study. Heart.

[17] Folsom AR, Kushi LH, Anderson KE (2000). Associations of general and abdominal obesity with multiple health outcomes in older women: the Iowa Women's Health Study. Arch Intern Med.

[18] Sabah KMN, Chowdhury AW, Khan HLR (2014). Body mass index and waist/height ratio for prediction of severity of coronary artery disease. BMC Res Notes.

[19] Kaur S, Sharma A, Singh HJ (2015). Waist-related anthropometric measures: Simple and useful predictors of coronary heart disease in women. Nat J Physiol Pharm Pharmacol.

[20] Lakka HM, Lakka TA, Tuomilehto J, Salonen JT (2002). Abdominal obesity is associated with increased risk of acute coronary events in men. Eur Heart J.

[21] Nishtar S, Wierzbicki AS, Lumb PJ (2004). Waist-hip ratio and low HDL predict the risk of coronary artery disease in Pakistanis. Curr Med Res Opin.

[22] Parsa AF, Jahanshahi B (2015). Is the relationship of body mass index to severity of coronary artery disease different from that of waist-to-hip ratio and severity of coronary artery disease? Paradoxical findings. Cardiovasc J Afr.

[23] Rashiti P, Behluli I, Bytyqi AR (2017). Assessment of the correlation between severity of coronary artery disease and waist–hip ratio. Open Access Maced J Med Sci.

[24] Zen V, Fuchs FD, Wainstein MV (2012). Neck circumference and central obesity are independent predictors of coronary artery disease in patients undergoing coronary angiography. Am J Cardiovasc Dis.

[25] Rubinshtein R, Halon DA, Jaffe R, Shahla J, Lewis BS (2006). Relation between obesity and severity of coronary artery disease in patients undergoing coronary angiography. Am J Cardiol.

[26] Niraj A, Pradahan J, Fakhry H, Veeranna V, Afonso L (2007). Severity of coronary artery disease in obese patients undergoing coronary angiography:“obesity paradox” revisited. Clin Cardiol.

[27] Morricone L, Ferrari M, Enrini R (1999). The role of central fat distribution in coronary artery disease in obesity: comparison of nondiabetic obese, diabetic obese, and normal weight subjects. Int J Obes Relat Metab Disord.

[28] Bays HE, González-Campoy JM, Bray GA (2008). Pathogenic potential of adipose tissue and metabolic consequences of adipocyte hypertrophy and increased visceral adiposity. Expert Rev Cardiovasc Ther.

[29] Bays H, Ballantyne C (2006). Adiposopathy: why do adiposity and obesity cause metabolic disease?. Future Lipidol.

[30] Bays HE (2011). Adiposopathy: is “sick fat” a cardiovascular disease?. J Am Coll Cardiol.

[31] Namazi N, Djalalinia S, Mahdavi-Gorabi A (2020). Association of wrist circumference with cardio-metabolic risk factors: a systematic review and meta-analysis. Eat Weight Disord.

[32] Hajsadeghi S, Firouzi A, Bahadoran P, Hassanzadeh M (2016). The value of wrist circumference for predicting the presence of coronary artery disease and metabolic syndrome. Indian Heart J.

[33] Eslami A, Mozaffary A, Derakhshan A, Azizi F, Khalili D, Hadaegh F (2017). Sex-specific incidence rates and risk factors of premature cardiovascular disease A long term follow up of the Tehran Lipid and Glucose Study. Int J Cardiol.

